# How Not to Diversify Philosophy of Religion: A Critique from the Twenty-First Century

**DOI:** 10.1007/s11841-024-01007-z

**Published:** 2024-03-06

**Authors:** Rafal K. Stepien

**Affiliations:** Institute for the Cultural and Intellectual History of Asia, Austrian Academy of Sciences, Vienna, Austria

**Keywords:** Philosophy of religion, ‘Eastern’ philosophy of religion, Cross-cultural philosophy of religion

## Abstract

Philosophy of religion has been the object of penetrating critiques concerning its continued near-complete blindness to all but a single religion. The need for philosophy of religion to open up so as to include more than merely occasional and tokenistic treatments of ‘Other’ religions is clearly evident from the slew of recently published titles concerned with diversifying the field. In this light, a book such as Victoria Harrison’s *Eastern Philosophy of Religion* should surely come as a welcome addition. And yet, unfortunately, this book turns out to be a case study in how *not* to diversify philosophy of religion in the twenty-first century.

There can be no doubting that philosophy of religion is in flux. Indeed, according to many, the field is in crisis. Above all, philosophy of religion has been, over the course of recent years, the object of penetrating critiques concerning its continued near-complete blindness to all but a single religion. This, coupled with a general suspicion of religious topics on the part of the naturalists who comprise the preponderant mass of contemporary analytic philosophers, has meant that philosophy of religion finds itself today segmented into many camps. While some philosophers of religion may still rally to traditional Christian themes in denial or dismissal of diversifist calls, and others may still assay efforts to render religious concepts innocuously naturalized, I believe it is safe to say, at the cusp of the second quarter of the twenty-first century, that philosophers of religion by and large acknowledge that change is needed.

The need for philosophy of religion to open up so as to include more than merely occasional and tokenistic treatments of ‘Other’ religions is clearly evident from the slew of recently published titles in one way or another concerned with diversifying the field. These, for example, task themselves with *Engaging Philosophies of Religion: Thinking Across Boundaries* (Bilimoria et al., 2024), *Diversifying Philosophy of Religion* (Loewen & Rostalska, 2023), *Renewing Philosophy of Religion* (Draper & Schellenberg, 2017), providing *Reconfigurations of Philosophy of Religion* (Kanaris, 2018), and envisioning *A Radical Pluralist Philosophy of Religion* (Burley, 2020). Others warn of *The End of Philosophy of Religion* (Trakakis, 2008), attempt a *Postcolonial Philosophy of Religion* (Bilimoria & Irvine, 2009), try to chart *The Future of the Philosophy of Religion* (DuJardin et al., 2021), or conceive *Religious Philosophy as Multidisciplinary Comparative Inquiry* (Wildman, 2010), or consider *Philosophies of Religion: A Global and Critical Introduction* (Knepper, 2022). I myself have previously reviewed the state of the field and such varied attempts to revive it in the course of proffering my own ‘Prolegomena to a Buddhist Philosophy of Religion’ (Stepien, 2023), and so there is no need for me to go into further details on these accounts here. What deserves stating is that, given the thrust of these publications, a book such as Victoria Harrison’s *Eastern Philosophy of Religion* (Harrison, 2022) should surely come as a welcome addition: another endeavour working towards diversifying the field, rendering it fit for purpose in the globalized world we live in today. And yet, unfortunately, this book turns out to be marred not only by some of the theoretical and methodological problems revisionist works such as those just mentioned overtly seek to move beyond, but also by many problems of detail distinctly its own.

In turning to review Harrison’s work directly, I feel it behoves me to make a full disclosure first off: I was a blind peer reviewer for the manuscript of this book. In fact, my opening remarks to the series editor Yujin Nagasawa at that stage were as follows:

In reviewing this work, I have been led by the material to take the following as my two guiding questions. Firstly, is the project, as currently conceived, valid, and hence worthy of publication? And secondly, irrespective of the validity of the project as assessed according to external criteria, is the project in its own terms, as currently executed, well done, and hence worthy of publication? Unfortunately, I have come to the conclusion that the answer is ‘no’ in both cases. I therefore do not recommend this work for publication.

Evidently, the work was published despite this assessment (and without being sent back subsequently for any review of revisions). But although some of the most egregious faults I pointed out in the manuscript were ironed out of the book, the published work nevertheless remains largely unchanged. Unfortunately, then, my overall view is that the book is so incorrigibly deficient that it did not merit being published. My detailed comments below may be taken as substantiation of this assessment.

Let me turn firstly to the question of whether the overall project this book undertakes is merited. In brief, I submit that it is no longer valid, today, to publish scholarly material on ‘Eastern’ traditions, and certainly not under the banner of a university press so highly regarded as Cambridge. This is not simply a gripe with the wording of the title. It is, rather, a conviction that to attempt, as Harrison has here done, to cover ‘Eastern’ (or ‘Asian’, or ‘Oriental’…) traditions in summary manner, is, in the twenty-first as opposed to the nineteenth century, intellectually irresponsible (more on this below). For apart from anything else, it reinforces the notion that all these traditions can be meaningfully covered under a single rubric (it is not worth distinguishing them properly) and in a single volume (it is not worth devoting individual treatments to them).

My worry to this effect is only reinforced by the fact that Harrison has decided to cover no less than the ‘highly diverse philosophies of Indian and Chinese Buddhism, Hinduism, and Jainism’ (Abstract) in a single slim text of just 62 pages. Indeed, as if that were not enough, she also includes discussions of the *Yijing*, Daoism, and (Neo-)Confucianism. Harrison has pursued this strategy despite acknowledging that ‘religions in Asia are generally not perceived to be sufficiently like one another to merit common treatment’ (4).^1^ To this view, she understandably feels a need to respond, and does so with a claim that, despite being ‘highly diverse’, the traditions she has placed within her covers are nevertheless somehow sufficiently unified to justify a blanket term such as ‘Eastern Philosophy of Religion’ because ‘Conceptually, this contrast [“with its Western counterpart”] makes sense’ (3). In support of this claim, however, all Harrison can muster is a statement averring that the distinction makes sense because ‘Western philosophy of religion refers to philosophy concerned with Western religions… while Eastern philosophy of religion is philosophy targeted on the philosophical dimensions of the religions of Asia’ (3). Apart from the fact that this claim is straightforwardly tautologous (Eastern is not Western because it is Eastern; Western is not Eastern because it is Western), I am at a complete loss to understand quite how it is supposed to support a conceptual—as opposed to a purely geographical—difference.

Indeed, in response to the claim that ‘the distinction between Eastern and Western philosophy of religion’ (5–6) ‘Conceptually… makes sense’ (3), I can only aver that the idea that ‘East is East and West is West’, as it were, is one of colonial-era heritage, and indelibly imprinted with the tremendous prejudices of that era. It is, therefore, frankly deeply troubling to find it still endorsed here. While Harrison may refer, in passing, to scholarship designed to render ‘the contrast between supposedly “Western” and “Eastern” philosophy of religion… obsolete’ (3, cf. 5–6), it is unfortunately true that this work cannot be classed within that category of works actively engaged in subverting relevant dichotomies. On the contrary. As such, despite Harrison’s efforts at justification, the treatment of these traditions in this book is irredeemably ‘anachronistic’ (6).

Now, to be fair, Harrison admits at the outset, thus:

The topic of Eastern philosophy of religion is potentially so expansive that the task of addressing it within a small Element such as this one could be compared to the task of doing the same with the topic of Western civilization! The reader should be warned that an attempt such as this can only be intrepid, and that what is presented here is the author’s selective view on which philosophical ideas and debates have decisively shaped Asian spiritual traditions. (2)

Any treatment, even the most expansive, will be selective, and this will necessarily be all the more so in a small book format such as the Elements series. And it is indubitably true that, while Harrison is clearly convinced of the value of Eastern philosophical traditions, the field of philosophy of religion as a whole remains stubbornly disproportionately focussed on Christian modes of thought. This is so despite the fact that, as Harrison herself asserts, the ‘most exciting development within academic philosophy of religion within recent decades’ may well have been ‘the trend towards expanding the subject area of the discipline to reach beyond its traditional focus on arguments and ideas connected to Western theism’ (60). (That said, Harrison does not cite a single one of the works mentioned above, leading one to wonder whether her passing reference to this trend is actually grounded in any serious engagement.)

As such, there is certainly need—pressing need—for works which do serve to open up philosophy of religion to hitherto ignored and/or sidelined traditions and perspectives. As a particularly relevant case in point, the ‘Elements in the Philosophy of Religion’ series itself has hitherto published 31 titles, and this is the single one devoted to ‘Eastern’ traditions. It cannot be denied, therefore, that the series, and the field at large, stands in need of alternative perspectives. But unfortunately, in order to aid in the broader project of opening up philosophy of religion, much more is needed than a single ‘token’ book to cover all and sundry ‘Other’ materials. If the series can justify literally dozens of theism-specific, and even overtly Christianity-specific, titles, then surely to limit practically everything else to this single volume smacks of precisely the kind of Christianocentric bias philosophy of religion routinely stands accused of.

Of course, my point as to the need for several titles rather than a single token one is a matter for the series editor, but it is relevant here in support of my overall point as to the unviability of the project as here conceived. I would suggest that, if Harrison does insist on pursuing a study of ‘Eastern Philosophy of Religion’, then at the very least she devote sufficient space to each of the traditions she treats in a manner that is not hopelessly tokenistic. This would of course entail writing a much weightier book, or books, but that could only be a gain on this amalgam of dismally desultory treatments.

The problem of coverage is compounded by the fact that the treatments of Hindu, Daoist, Confucian, and Jain (as well as Yogācāra and Chan/Zen Buddhist) ideas in Harrison’s work are so cursory as to be hindrances rather than aids to understanding. While a thematic approach is welcome, I submit that to pursue a thematic approach while attempting to cover not just one entire religious/philosophical tradition (already an impossibly gargantuan task) but some half a dozen in the confines of one book (*a fortiori* a tiny one) is imprudent to say the least. Nor can I understand how treating one or another tradition (as opposed to several of them lumped together) ‘would inevitably result in a loss of philosophical depth in a short work such as this one’ (4). Surely, obviously, the contrary is true, for how can depth be gained by treating multiple traditions superficially? I say this on the understanding, of course, that there is nothing stopping an author writing an account of Hindu, Daoist, Confucian, Jain, or Buddhist philosophy of religion, say, from mentioning inter-religious influences while nevertheless providing a focussed account of that singular tradition’s positions and arguments.

This ties in with another, methodologically oriented, concern as to the book as a whole. Although Harrison positions her work within ‘Global Philosophy’ (61–62), I would argue that a book such as this, ‘largely the result of the author’s grounding in the methods of analytic philosophy’ (62) and *not*, as such, the result of expertise in any of the Asian traditions under study, only serves to reinforce, rather than undermine, the presumptuous yet still common idea that ‘Eastern’ traditions are sufficiently *un*sophisticated as to warrant treatment by anyone sufficiently trained in ‘real’ (that is, Western) philosophy or philosophy of religion.

Moving on, in her attempt to anchor the work in some semblance of coherence, Harrison states: ‘this Element demonstrates that the religious philosophies of Asia, while not focused on a common concept, such as the concept of God, have several overlapping concerns’ (5). Apart from the perplexing reference to God, implying as it seems to do that ‘the religious philosophies of Asia’—whatever these are—are not concerned with the concept of God (an implied claim both sweeping and wrong), the immediate problem with this idea is that it actually refers to ‘overlapping concerns’ of such utter generality that *any* philosophical/religious tradition under the sun could reasonably be included. After all, which religious/philosophical tradition has not dealt with those topics Harrison explicitly stipulates: ‘being, non-being, and becoming’ (5)?

And yet, the problem transpires to be still worse, for there are multiple occasions where Harrison attempts to identify a link between diverse Asian traditions or ideas that simply does not withstand analysis, be it historical or analytical, and that therefore gives the distinct impression of being forced upon the materials so as to justify her approach. Thus, for example, Harrison speaks of an ‘extraordinary convergence… It is remarkable that, in the early centuries of the Common Era, philosophers in both China and India were grappling with the fundamental ontological question of how nothing/emptiness and something/form come into relation to constitute both the world that we experience and ourselves as the experiencers of that world’ (49). But one could say the same thing about every philosopher from Plato to Anselm to Leibniz to Kant to Sartre… so the notion that this constitutes an ‘extraordinary convergence’ is overblown to say the least, if not downright disingenuous.

Another example in this vein is when Harrison claims that

The conceptual connection between nothing and empty space became one of the foundational assumptions for many different approaches both to cosmology and to spiritual practice within East Asia. It quickly led, for instance, to the high valuation of empty spaces (*kong* 空) that eventually, after the passage of many centuries, inspired the remarkable, austere aesthetic tradition of Zen Buddhism. (49)

This is quite a leap! Especially since close to a full millennium separates the early Daoist ideas of the *Dao De Jing* Harrison is discussing from the Chan Buddhist aesthetic she refers to, which dates from Tang-dynasty China at the earliest, if not from still later developments of Zen in Japan. How can it have ‘quickly led,’ then, to developments only evident ‘after the passage of many centuries’? To make this particular example still more egregious, however, Harrison then states ‘This development within Zen no doubt also built on the connection between space and meditation made in several verses within the Pāli canon’ (49), which is as tenuous a link as it is vacuous.

Turning to the quality of the work within the terms of the project itself, as stated above this too unfortunately does not stand up to scrutiny. For one thing, there are some highly problematic generalizations. For example, Harrison declares that

religious philosophy quickly assumed far greater prominence in India than it did in traditional Chinese, Japanese (De Bary et al. 2001), or Korean (Lee & De Bary 1997) thought. On the Indian sub-continent, philosophers were concerned with religious questions to a degree not found elsewhere in Asia (3).

This is the kind of uninformed, uncontextualized, unjustified, and essentializing declaration I would mark down in an undergraduate essay; one I would certainly not expect in a scholarly book published by a reputable press. Apart from anything else, as a specialist of both Indian and Chinese Buddhist philosophy myself, I honestly have no idea what Harrison could possibly mean by these statements. To put it in as profound an understatement as I am capable of, there was, and is, certainly no shortage of religious philosophizing in China, or Japan, or Korea! So I am left to conclude that Harrison’s claim rests on blithe stereotyping or plain ignorance (or a combination of both), and is inadmissible on either count. Besides which, if Harrison is going to make wildly over-arching claims about differing degrees of religiosity among the philosophies of Asia, the least she can do is explain what she means by ‘religion’ and ‘philosophy’. After all, unquestioningly imposing these categories as they are used (in English) today only serves to continue provincializing, rather than de-provincializing, the East.

At least Harrison makes an attempt at ‘Problematizing “Religion”’ (5). But the single paragraph she devotes to this matter is not only hopelessly cursory but does not help matters at all. Indeed, apart from the fact that it only refers in passing to one of Harrison’s prior articles, the reasons for treating Asian religions as religions which Harrison does adduce do not relate to why such traditions should (or should not) be treated under the rubric of ‘philosophy of religion’, to say nothing of how their distinctive identities serve to modify standard conceptions of what this is and is about. As such, it is not at all clear how Harrison even conceives of the very foundational terms of her study, and nor is it at all clear how she conceives her sources to have conceived equivalent or analogous terms in their own languages.

Speaking of languages, it is certainly noteworthy that the bibliography includes no sources at all in any of the original languages (indeed, no sources in any language other than English). From what is presented in the work itself, therefore, it appears that Harrison is not conversant with any of the languages in which the original texts she studies were composed. Why should this be acceptable? After all, it would certainly not be acceptable for a scholar of ancient Christianity, say, to not know any of the relevant classical languages (Hebrew, Greek, Latin…), or for a scholar of Continental philosophy of religion, say, to not know German or French. As such, I find that Harrison’s acknowledged wholesale reliance on modern translations and works of secondary scholarship (and these in English alone) renders her insufficiently expert to write with the requisite authority on any of the traditions she surveys. Perhaps still more concerningly, I find that the willingness to write on ‘Eastern’ traditions without adequate training is indicative of the prejudice I referred to above, according to which such exotic Oriental Others are implicitly deemed sufficiently simple such that just about anyone can cobble together a book on them. All told then, in the absence of any evidence to the contrary, I find Harrison’s presuming to undertake this project on this basis to be intellectually irresponsible.

My concern as to authorial inexpertise is exacerbated by the presence in the bibliography of some translations that no-one considers reliable today (e.g., Jacobi: from 1884!) and of numerous translations taken from omnibus compendia (e.g., all the Sourcebooks, Handbooks, and related works). There is also a preponderance of introductory texts among the works cited (examples too many to mention), all of which reinforces the distinct impression that Harrison is not conversant with relevant specialist scholarship and is therefore not qualified to write of the traditions and materials she has undertaken to.

Finally, another point to consider in assessing whether this book even merited publishing is the degree to which it reproduces Harrison’s prior work. Harrison refers several times to her (much longer) book *Eastern Philosophy: The Basics* (Harrison, 2013; 2nd ed. 2019), and in fact there are several marked correspondences, for example in her treatments in both works of Jain perspectival pluralism (Harrison, 2022: 57–59; 2013: 42–46), Śaṅkara (Harrison, 2022: 39–42; 2013: 56–60), Nāgārjuna (Harrison, 2022: 32–33; 2013: 97–98), Zhu Xi (Harrison, 2022: 54; 2013: 168–170), and Wang Yangming (Harrison, 2022: 55; 2013: 172–174). To some extent, this is inevitable when introducing the same thinkers and ideas again, and I am not accusing Harrison of self-plagiarism, but it is nonetheless evident that substantial overlap exists between her treatments of several figures and themes in both works.

In conclusion then, it should be clear that there is no reason why I would recommend this book. Not only does it contain nothing that pushes the frontiers of any field of knowledge further, but everything it says is said better elsewhere. Perhaps, then, it may stand as a model of how *not* to diversify philosophy of religion in the twenty-first century.

## Footnotes

1 All unreferenced page numbers refer to Harrison 2022.
